# Hyperphosphatemia Promotes Senescence of Myoblasts by Impairing Autophagy Through Ilk Overexpression, A Possible Mechanism Involved in Sarcopenia

**DOI:** 10.14336/AD.2017.1214

**Published:** 2018-10-01

**Authors:** Patricia Sosa, Elena Alcalde-Estevez, Patricia Plaza, Nuria Troyano, Cristina Alonso, Laura Martínez-Arias, Andresa Evelem de Melo Aroeira, Diego Rodriguez-Puyol, Gemma Olmos, Susana López-Ongil, María P. Ruíz-Torres

**Affiliations:** ^1^System Biology Department, Alcala University, Alcalá de Henares, Madrid, Spain.; ^2^Research Unit, Biomedical Research Foundation from Príncipe de Asturias University Hospital, Alcalá de Henares, Madrid, Spain.; ^3^Geriatric and Frailty Section, Getafe University Hospital, Getafe, Madrid, Spain.; ^4^Bone and Mineral Research Unit, Hospital Universitario Central de Asturias. Instituto de Investigación Sanitaria del Principado de Asturias, Red de Investigación Renal (REDinREN] del ISCIII, Oviedo, Spain; ^5^Nephrology Section, Biomedical Research Foundation from Principe de Asturias University Hospital, Alcalá de Henares, Madrid, Spain; ^6^Instituto Reina Sofía de Investigación Nefrológica, IRSIN, Madrid, Spain.

**Keywords:** hyperphosphatemia, senescence, myoblasts, ILK, autophagy, sarcopenia

## Abstract

In mammalians, advancing age is associated with sarcopenia, the progressive and involuntary loss of muscle mass and strength. Hyperphosphatemia is an aging-related condition involved in several pathologies. The aim of this work was to assess whether hyperphosphatemia plays a role in the age-related loss of mass muscle and strength by inducing cellular senescence in murine myoblasts and to explore the intracellular mechanism involved in this effect. Cultured mouse C_2_C_12_ cells were treated with 10 mM beta-glycerophosphate (BGP] at different periods of time to induce hyperphosphatemia. BGP promoted cellular senescence after 24 h of treatment, assessed by the increased expression of p53, acetylated-p53 and p21 and senescence associated β-galactosidase activity. In parallel, BGP increased ILK expression and activity, followed by mTOR activation and autophagy reduction. Knocking-down ILK expression increased autophagy and protected cells from senescence induced by hyperphosphatemia. BGP also reduced the proliferative capacity of cultured myoblasts. Old mice (24-months-old] presented higher serum phosphate concentration, lower forelimb strength, higher expression of p53 and ILK and less autophagy in vastus muscle than young mice (5-months-old]. In conclusion, we propose that hyperphosphatemia induces senescence in cultured myoblasts through ILK overexpression, reducing their proliferative capacity, which could be a mechanism involved in the development of sarcopenia, since old mice showed loss of muscular strength correlated with high serum phosphate concentration and increased levels of ILK and p53.

Aging is a multifactorial phenomenon characterized by the progressive deterioration of physiological functions, which increases the susceptibility of the organism to many diseases and death [1]. In mammalians, advancing age is associated with sarcopenia, the progressive and involuntary loss of muscle mass and strength [2]. Sarcopenia reduces independence and the quality of life for individuals, and leads to falls and fractures with escalating health costs for the rapidly aging human population. The sarcopenic muscle is characterized by atrophy of type II fibers, changes and heterogeneity in fiber size, accumulation of fat and connective tissue between the fibers, decline in mitochondrial metabolism and in its oxidative capacity, increased inflammation and protein catabolism [3, 4]. Sarcopenia is also related to a reduction in the number and function of satellite cells, the myogenic stem cells, inside fibers, which can compromise the regenerative capacity of the aged muscle [5). In resting muscle, satellite cells are quiescent and become activate after injury or growth stimulus. Activated satellite cells, which are called myoblast, proliferate massively to generate the myogenic progenitors needed for muscle regeneration or can return to a quiescent state to maintain the satellite cell pool. In this case, cell fate decisions are regulated by intrinsic or extrinsic factors present in the microenvironment of the satellite cell niche [6). The intracellular mechanism underlying the progressive loss of muscle function has not been completely elucidated yet, and hence, there has been a growing interest in the study of the factors that play a role in the genesis of sarcopenia in order to develop new therapeutic interventions able to reduce or to slow down age-associated muscle wasting [7).

At cellular level, aging is known as cellular senescence, defined by Hayflick as an irreversible arrest of cell division [8). Cells come into senescence after a determined number of divisions by the telomere shortening [9], or prematurely triggered by stressful stimuli, such as activated oncogenes, DNA damage, or oxidative stress [10-12]. Senescent cells have an altered cellular morphology, increased activity of senescence-associated-β-galactosidase (SA-β-Gal), accumulation of DNA damage foci, induction of senescence associated secretory phenotype (SASP) and loss of proteostasis [13]. With age and in some premature aging syndromes, the number of senescent cells increases in multiple tissues, disrupting tissue homeostasis and contributing to its age-related dysfunction [14].

Cellular senescence is induced in different cellular types by changes in several environmental and systemic factors. Among others, hiperosmolarity, high glucose [15], glycated albumin [16] and hyperphosphatemia [17] have been described as inductors of cellular senescence. Recent studies have analyzed the role of elevated serum phosphate concentration in aging-related pathologies [18]. Regulation of the serum phosphate level is dependent on dietary phosphate intake, intestinal absorption and renal excretion. Renal and intestinal management of phosphate is mainly regulated by PTH, Vitamin D, the Fibroblast Growth Factor 23 (FGF23), and Klotho [19]. Mice lacking Klotho or FGF23 exhibit a premature aging syndrome associated with abnormal mineral metabolism characterized by hyperphosphatemia, hypercalcemia, and hypervitaminosis D [20]. Several genetic and dietary interventions to reduce serum phosphate rescue the premature aging syndrome, suggesting a primary role of hyperphosphatemia in premature aging. The effect of high extracellular phosphate concentration has been studied in several cell types. Recent studies suggest that it induces changes in cellular phenotype, including osteoblast mineralization and differentiation [21], osteaoclast differentiation [22] and calcification of vascular smooth muscle [23]. Since the FGF23 KO mice and the Klotho mice exhibited severe muscle wasting [20], we wondered whether hyperphosphatemia could directly affect the skeletal muscle cells, leading to sarcopenia.

Besides, cellular senescence is regulated by conserved intracellular signaling pathways and an emerging role of integrin linked kinase (ILK) in the aging process has been recently described [24]. ILK is a component of the multiprotein transmembrane complex mediating integrin-dependent signalling. ILK has serine-threonine kinase activity and it links the actin cytoskeleton and other intracellular signalling pathways with the extracellular matrix [25, 26]. ILK regulates proliferation, migration and other cellular functions, but only a few works describe a relationship with the aging process. Increased expression of ILK and fibronectin and β1 integrin expression have been described in old rats [27]. In cultured cells, ILK overexpression induced senescence-associated alteration in aging cardiac fibroblasts [28], whereas genetic reduction of ILK in C. elegans and Drosophyla extended lifespan [29]. Also, we have recently demonstrated that ILK overexpression reduced the expression of Klotho in cultured renal cells [30].

The aim of the present work was to assess whether hyperphosphatemia plays a role in the age-related loss of mass muscle and strength by inducing cellular senescence in murine myoblasts and to explore the intracellular mechanism involved in this effect.

## MATERIALS AND METHODS

### Materials

Culture media Dulbecco’s modified Eagle’s medium (DMEM) was from Lonza (Basel, Switzerland). Foetal bovine serum (FBS), antibiotics, OptiMEM and anti-GAPDH antibody were from Life Technologies Ltd., (Carlsbad, CA, USA). Culture plates and blueStar-prestained protein marker were from Cultek (Thermo Fisher Scientific brand, Madrid, Spain), and Chemiluminescence reagent detection system was from Pierce (Thermo Fisher Scientific brand, Madrid, Spain). The fluorogenic ImaGene green C_12_FDG (5-dodecanoyl-aminofluorescein di-ß-D-galactopyranoside) substrate reagent and ProLong® Gold antifade reagent with DAPI were from Molecular Probes (Thermo Fisher Scientific brand, Madrid, Spain). Acrylamide-bisacrylamide was from Hispanlab-Pronadisa (Madrid, Spain). Protein Assay for measurement protein concentration, electrophoresis equipment and PVDF membrane were from Bio-Rad Laboratories (Richmond, CA, USA). Protease inhibitor cocktail tablets and 5-Bromo-2-deoxy-uridine (BrdU) were from Roche Diagnostics S.L. (Barcelona, Spain). Fluorescein isothiocyanate (FITC)-conjugated anti-BrdU was from Becton-Dickinson (San Jose, CA, USA). The secondary horseradish peroxidase-conjugated goat antimouse IgG and antirabbit IgG was from Dako (Glostrup, Denmark). Antibodies against phospho-GSK3β (Ser9), GSK3β, ILK1, acetyl-p53 (Lys382), phospho-S6 Ribosomal protein (Ser235/236), S6 Ribosomal protein, SQSTM1/p62 and LC3B were from Cell Signalling Technology (IZASA, Barcelona, Spain). Anti-PCNA (PC10), small RNA interference (siRNA) against ILK and Silencer negative control (scrambled) were from Santa Cruz Biotech (Sta. Cruz, CA, USA). Anti-p21 antibody was from BD Biosciences (Erembodegem, Belgium). Anti-myosin heavy chain (MHC) was from R&D System (Minneapolis, MN, USA) and desmin was from Abcam (Cambridge, UK). QuantiChrom phosphate assay kit (DIPI-500) was from Bioassay System (Hayward, CA, USA). Metafectene transfection reagent was from Biontex Laboratories (Munich, Germany). Beta-glycerophosphate (BGP), rapamycin, phosphonoformic acid (PFA), anti-p53 antibody, chloroquine, and the rest of drugs or reagents (unless otherwise indicated) were from Sigma-Aldrich-Fluka Chemical Co. (St. Louis, MO, USA).

### Cell culture

C_2_C_12_, a mouse myoblast cell line, was from American Type Culture Collection (Manassas, VA, USA). Cells were grown in Dulbecco’s Modified Eagle Media (DMEM) containing 4.5 g/L glucose and supplemented with 10% FBS, 100 U/mL penicillin and 100 µg/mL streptomycin in an atmosphere of 95% air and 5% CO_2_. Cells were used at passages 3-10.

### Animal studies

Five- and twenty-four-month-old male C57BL6 mice were obtained from Janvier Laboratories. Animals were kept on a 12:12 h light-dark cycle, at 24ºC, and food and water were available ad libitum. Then, mice were anesthetized, and blood samples were collected by heart puncture exsanguinations. Skeletal muscle was obtained and conserved in RNA later solution for protein extraction. Serum phosphate was measured by a commercial kit according to manufacturer instructions.

The study design and the experimental protocols were performed in agreement with the Guide for the Care and Use of Laboratory Animals published by the US National Institute of Health (NIH Publication No.85-23, revised 1996) and with the European Union regulations (EU Directive 2010/63/EU). The study was revised and approved in accordance with the Ethics Committee from Alcala University for mice (Madrid, Spain).

### Forelimb grip strength test

To measure forelimb grip strength, we used a Grip Strength Meter (UGO BASILE) from PSYMTEC (Madrid, SPAIN). The procedure was as follows: grip strength was measured by gently pulling the mouse by the tail in a horizontal plane parallel with the base plate of the grip strength meter. The animal’s grip strength was measured as peak force registered. Each mouse was tested ten times consecutively per day at one-minute intervals, with the maximum grip strength used in analyses. The order of mice tested by this test was randomized. All investigators were blinded to all results. Test sessions were performed during the afternoon hours of the light cycle (11 AM to 14 PM).

### Protein extraction and immunoblot analysis

After treatments, cells were washed twice with cold PBS and lysed in buffer (10 mM Tris-HCl pH 7.4, 1 mM EDTA, 1% Triton X-100, 0.1% sodium deoxycholate) containing a protease inhibitor cocktail. The resulting solution was spun at 13,000 rpm for 30 min at 4 ºC. Protein concentration was determined with BioRad protein assay kit. Proteins samples were run onto 8-12% SDS-polyacrylamide gels (PAGE) under reducing conditions and then transferred onto PVDF membranes. Membranes were blocked with 5% non-fat milk in Tween Tris buffered saline (TTBS) (20mM Trizma, pH 7.6, 150mM NaCl, 0.05% Tween-20) for 1 h at room temperature (R/T), and incubated with the corresponding primary antibodies overnight at 4ªC and then incubated with secondary antibodies at room temperature for 1 h. The immunoreactive bands were visualized with the chemiluminescence reagent detection system and densitometrically analysed by using Image J software 2.6. Then, blots were reblotted with a mouse anti-GAPDH antibody in order to normalize protein levels.

### Quantitative RT-PCR

Total RNA from C_2_C_12_ cells was isolated using TRIzol reagents according to the manufacturer’s protocol. cDNA was synthesized using a High-Capacity cDNA reverse transcription kit (Applied Biosystems Inc., Foster City, CA, USA), the gene expression was measured by quantitative PCR (ABI Prism 7500 Fast Real-Time PCR System) and analyzed with 7500 Fast sequence detection software v1.3.1 (Applied Biosystems Inc., Foster City, CA, USA), using TaqMan and SYBR green genes and Double delta Ct method. TaqMan genes: MCP1 (Mm00441242_m1) and the endogenous control beta-actin (Mm00607939_s1). SYBR green primers: IL6 (from 5 to 3), forward: CCG GAG AGG AGA CTT CAC AGA GGA, and reverse: AGC CTC CGA CTT GTG AAG TGG TAT A; TNFα (from 5 to 3), forward: TGG CCC AGA CCC TCA CAC TCA, and reverse: GGC TCA GCC ACT CCA GCT GC and the endogenous control glyceraldehyde-6-phosphate dehydrogenase (GAPDH) (from 5 to 3), forward: CCA CCC AGA AGA CTG TGG AT, and reverse: CACATT GGG GGT AGG AAC AC.

### Detection of senescence-associated ß-galactosidase activity by fluorescence confocal microscopy

C_2_C_12_ cells were grown in microscope cover glasses and after 24 h of free-serum DMEM, they were treated with BGP, at different times, in the presence or not of different antagonists. To determine cellular senescence, SA-ß-GAL activity was measured by fluorescence confocal microscopy, using the fluorogenic substrate C_12_FDG (Dimri *et al*., 1995). After BGP treatment, 33 µM C_12_FDG was added for 4 h. At the end of incubation, cells were washed twice with PBS, and fixed with 4% para-formaldehyde for 15 min. Subsequently, cells were washed again and mounting in ProLong® Gold antifade reagent with DAPI overnight. Samples were analyzed using LEICA TCS-SP5 confocal microscope (Leica Microsystems; GmbH, Mannheim, Germany) at 488 nm argon laser to detect green fluorescence of SA-ß-GAL activity and at 405 nm to detect DAPI. Pictures were obtained, and fluorescence intensity was measured by densitometry by Image J software (http://rsbweb.nih.gov/ij/).

### C_2_C_12_ differentiation assay

Cells were grown in microscope cover glasses during seven days with 2% horse serum to promote myogenic differentiation, in the presence or the absence of 10 mM BGP.

To determine myotube formation, myosin heavy chain (MHC) and desmin expression were assessed by immunofluorescence using a confocal microscopy. After BGP treatment, cells were washed twice with PBS, and fixed with 4% paraformaldehyde for 15 min at R/T, then 0.5% Triton X-100 was added for 10 min at R/T. After that, cells were blocked with 5% BSA for 1 h at R/T, and then incubated first, with rabbit anti-desmin (1:500) overnight in a humid chamber at 4°C, and second, with mouse anti-MHC (1:100) for 2 h at R/T. After being washed in PBS, cells were incubated 1 h with a mix of 200-fold diluted goat anti-rabbit IgG labelled with Alexa Fluor 488 to detect desmin in green and 200-fold diluted goat anti-mouse IgG labelled with Alexa Fluor 647 to detect MHC in red. Finally, cover glasses were mounted on ProLong Gold antifade reagent with DAPI to stain nuclei in blue overnight. Preparations were visualized in confocal microscope LEICA TCS-SP5. Fluorescence intensity from pictures obtained was measured by Image J software.

### Transient transfection experiments

ILK was silenced in C_2_C_12_ cells by transfecting a specific small RNA interference (siRNA) against ILK (siILK, Santa Cruz). To knockdown expression of ILK, we used a mixture of three oligonucleotides ILK siRNA. An unspecific scramble was used as transfection control. Transfections were performed using the Metafectene reagent and OptiMEM for 24 h. After transfections, cells were incubated with complete DMEM for 24 h. After that, 10 mM BGP was added in some wells for 48 h using serum free DMEM. Afterward, cells were processed for checking ILK and, then, p53, p62 and pS6 protein expression, as described above.

### In vitro wound healing model

The monolayer of cultured cells C_2_C_12_ was scratched with a needle to give a 0.6 mm wide wound, washed and cultured in culture media with 10 mM BGP for 20 h or 24 h. Images of wound areas were captured with Moticam microscope (Motics Microscopes). The scratch wound area was measured using Image Plus Software, and percentage of wound closure at each time point was derived by the following formula [31]:

(1- [current wound size/initial wound size]) x100.

### Cell proliferation assay by BrdU incorporation

Cell proliferation was assessed by BrdU incorporation into cellular DNA according to the manufacturer’s instructions. After cell treatment, BrdU was added for the following 24 h. The level of BrdU cell incorporation was detected in cells labelled with propidium iodine and FITC-conjugated anti-BrdU monoclonal antibodies, analyzed at 488 nm on a FACScan flow cytometer (Becton-Dickinson).

**Figure 1.**
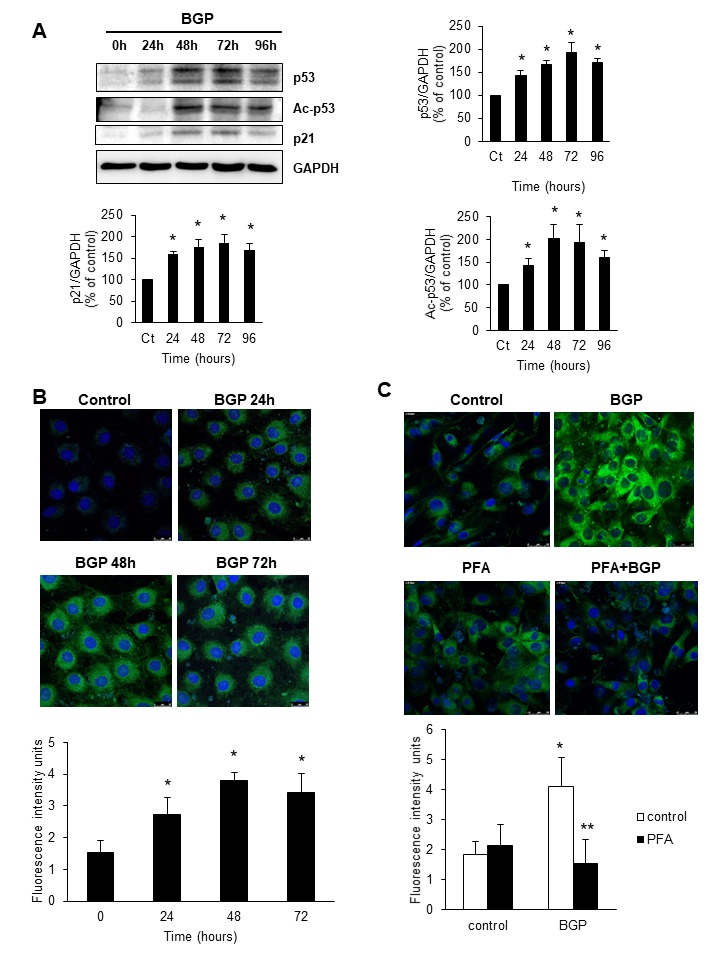
Hyperphosphatemia induces senescence in C_2_C_12_ cells Cultured C_2_C_12_ were treated with 10 mM BGP for 24, 48, 72 and 96 hours. **A**) Acetylated-p53 (Ac-p53), p53 and p21 expression were analysed by western blot. A representative blot is shown in each case. Bar graphs represent the densitometric analysis of the bands. The results are expressed as a percentage of control cells (time 0h) and are the mean ± standard error of the mean from five different experiments. * p< 0.05 vs control). **B**) Senescence associated β-galactosidase activity was analysed by confocal microscopy using the fluorogenic substrate C_12_FDG after 24, 48 and 72 hours 10 mM BGP incubation. **C**) C_2_C_12_ were treated with 10 mM BGP or vehicle for 48 hours in the presence or the absence of phosphonoformic acid (0.5 mM, PFA) and senescence associated β-galactosidase activity was evaluated as above. In **B and C**) A representative experiment is shown in each case. Bar graphs represent the densitometric analysis of the fluorescence of 20 cells. Results are expressed as arbitrary fluorescence intensity units and are the mean ± standard error of the mean from five different experiments. * p< 0.05 vs control. ** p<0.05 vs BGP.

### Statistical analysis

Results are expressed as the mean ± standard error of the mean of an independent variable number of experiments detailed in figure captions and as a percentage of the control values. GraphPad Prism 5 and Stata Software were used for statistical analysis. The following statistical tests were used: one-way or two-way ANOVA followed by Dunnett test for all experiments but wound healing experiments which were analysed using a repeated measures multilevel lineal model with treatment, time and time by treatment interaction test. Experiments performed in animals were analysed by an unpaired t-test with Welch’s correction. The level of statistical significance was defined as p < 0.05.

**Figure 2.**
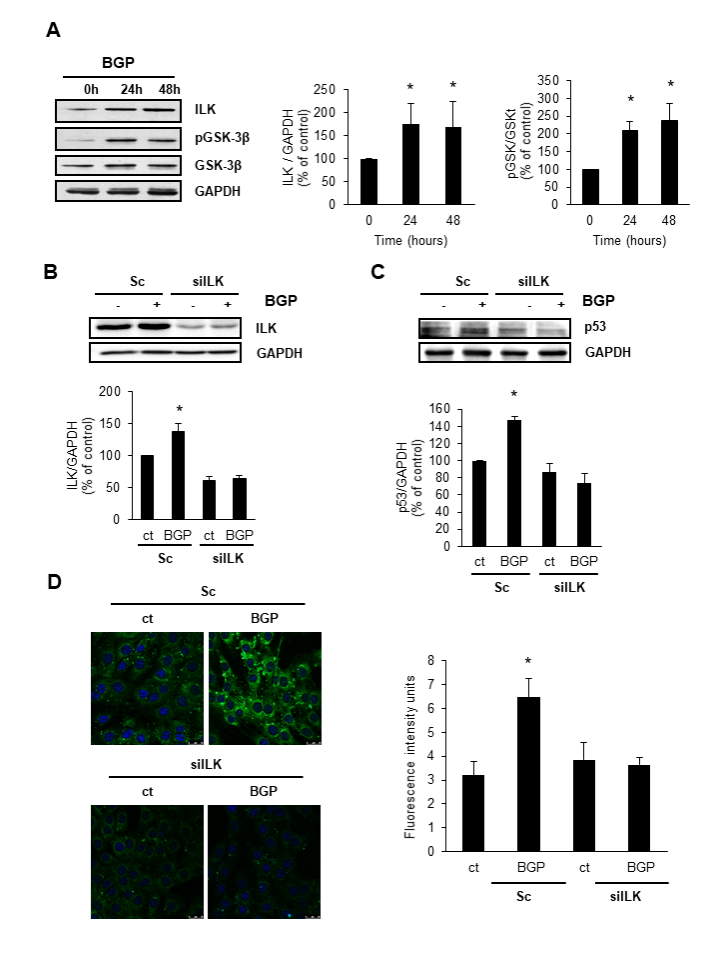
Hyperphosphatemia induces senescence by increasing ILK expression and activity **A)** C_2_C_12_ were treated with 10 mM BGP for 24 or 48 hours, then, ILK, phospho-GSK3β (pGSK-3ß) and total GSK3β were analysed by western blot. **B and C)** C_2_C_12_ were transfected with the specific siRNA against ILK (siILK) or unspecific siRNA as a control (scramble) and treated for 48 hours with 10 mM BGP. ILK **(B)** and p53 **(C)** expression was evaluated by western blot. A representative blot is shown in each case. Bar graphs represent the densitometric analysis of the bands. The results are expressed as a percentage of control and are the mean ± standard error of the mean from four different experiments. * p< 0.05 vs control. **D)** C_2_C_12_ were transfected with the specific siRNA against ILK (siILK) or unspecific siRNA as a control (scramble) and treated for 48 hours with 10 mM BGP. Senescence associated β-galactosidase activity was analysed by confocal microscopy using the fluorogenic substrate C_12_FDG. Bar graph represents the densitometric analysis of the fluorescence of 20 cells. Results are expressed as arbitrary fluorescence intensity units and are the mean ± standard error of the mean from four different experiments. * p< 0.05 vs control.

## RESULTS

### Hyperphosphatemia induces senescence in C_2_C_12_ cells by increasing ILK expression and activity

Cultured murine C_2_C_12_ cells were depleted of FBS during 24 h and then treated with 10 mM BGP as phosphate donor for different periods of time and the content of acetylated-p53, p53 and p21 was evaluated by western blot. BGP induced a significant increased expression of all proteins after 24 h of treatment (Fig. 1A) which remained elevated until 96 h of treatment. No changes were found in p16 expression (data not shown). Cellular senescence was also evaluated as SA-β-Gal staining by confocal microscopy. The fluorescent signal was increased 24 h after BGP addition and remained elevated until 72 h (Fig. 1B). We analysed the senescence associated secretory phenotype (SASP) of C_2_C_12_ cells after BGP treatment by testing mRNA expression of some pro-inflammatory cytokines such as IL-6, TNF-α and MCP-1 by RT-qPCR. Results showed a significant increase in the mRNA expression of IL-6, TNF-α and MCP-1 in a time-response way (Supplemental Fig. A, B and C, respectively). SA-β-Gal activity was also analysed after 48 h incubation of BGP in the presence or the absence of PFA, an inhibitor of the Na-Pi transporter (Pit-1). PFA blocked the senescence induced by BGP (Fig. 1C), suggesting a specific effect of phosphate on senescence.

Cells treated with 10 mM BGP increased ILK expression after 24 h of incubation, analysed by western blot. Also, the ILK activity, assessed by the increment in phosphorylation of GSK-3β, was increased without significant changes in the total GSK3β content (Fig. 2A). To establish a relationship between the increment in ILK and cellular senescence induced by BGP, we knocked down ILK expression using a specific RNA interference. After transfection with siILK, ILK expression was reduced by fifty per cent with respect to cells transfected with scramble RNA. Similarly, ILK expression did not increase after 48 h incubation with BGP (Fig. 2B). p53 expression was analysed in cells transfected with siILK or scramble and results showed that knocking down ILK prevented the BGP-induced increased expression of p53 after 48 h of treatment (Fig. 2C). Neither did SA-β-Gal activity increase after BGP treatment in C_2_C_12_ cells transfected with siILK (Fig. 2D). These results suggest that senescence induced by hyperphosphatemia was dependent on ILK overexpression.

**Figure 3.**
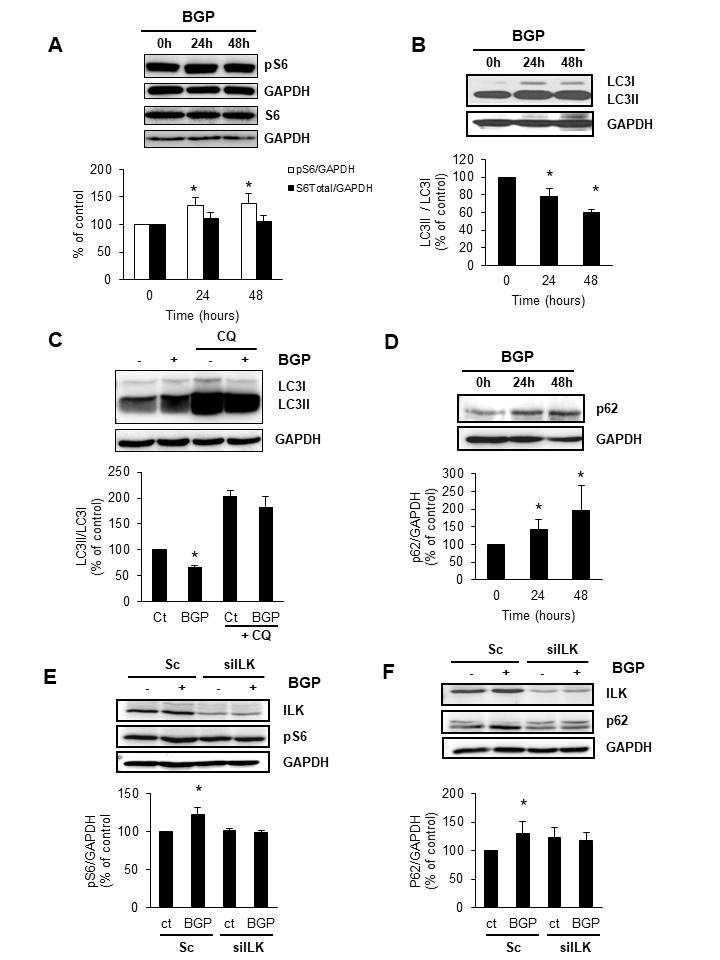
Hyperphosphatemia activates mTOR and reduces autophagy in myoblast through ILK activation **A, B and D)** C_2_C_12_ cells were incubated with 10 mM BGP for 24 or 48 hours, and with or without chloroquine (CQ, 20μM, 4h) (C). Phospho-S6 (pS6), the mTOR substrate, and total S6 (S6) **(A)**, ratio LC3II/LC3I (B and C) and p62 **(D)** protein expression were evaluated by western blot. A representative blot is shown in each case. Bar graphs represent the densitometric analysis of the bands. The results are expressed as a percentage of control and are the mean ± standard error of the mean from four different experiments. * p< 0.05 vs control. **E and F)** C_2_C_12_ were transfected with the specific siRNA against ILK (silLK) or unspecific siRNA as a control (Sc, scramble) and treated for 48 hours with 10 mM BGP. Then, ILK and pS6 **(E)** and ILK and p62 **(F)** were analysed by western blot. A representative blot is shown in each case. Bar graphs represent the densitometric analysis of the bands. The results are expressed as a percentage of control and are the mean ± standard error of the mean from five different experiments. * p< 0.05 vs control.

**Figure 4.**
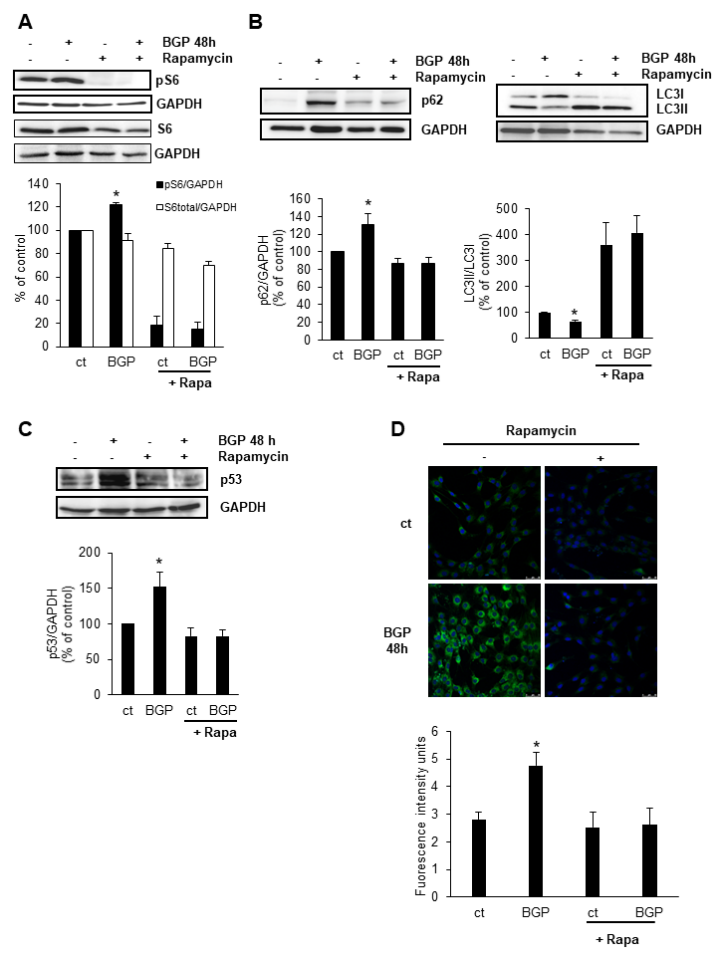
Inhibition of mTOR with rapamycin increases autophagy and protects myoblast from BGP-induced senescence C_2_C_12_ cells were treated with 10 mM BGP in the presence or the absence of 50 nM Rapamycin for 48 hours. Phospho-S6 expression (pS6) **(A)**, LC3II/LC3I ratio and p62 expression **(B)** and p53 expression **(C)** were analysed by western blot. A representative blot is shown in each case. Bar graphs represent the densitometric analysis of the bands. The results are expressed as a percentage of control and are the mean ± standard error of the mean from five different experiments. * p< 0.05 vs control. **D)** C_2_C_12_ were treated with 48 hours with 10 mM BGP in the presence or the absence of 50 nM rapamycin. Senescence associated β-galactosidase activity was analysed by confocal microscopy using the fluorogenic substrate C_12_FDG. Bar graph represents the densitometric analysis of the fluorescence of 20 cells. Results are expressed as arbitrary fluorescence intensity units are the mean ± standard error of the mean from four different experiments. *p< 0.05 vs control.

### Hyperphosphatemia activates mTOR and reduces autophagy in myoblast through ILK activation

To explore the intracellular mechanisms involved in senescence induced by hyperphosphatemia, we analysed the activation of mTOR and the autophagic flux in the presence of 10 mM BGP. Results showed that BGP increased mTOR activity significantly after 24 h of treatment without changes in total S6 content, assessed by the phosphorylation of S6 (Fig. 3A). Autophagy was measured by the ratio LC3II/LC3I. As presented in Fig. 3B, the ratio LC3II/LC3I was decreased after 24 h and remained until 48 h of BGP treatment. To analyse autophagic flux we analysed not only the ratio LC3II/LC3I in the presence or the absence of chloroquine (20 μM, 4 h), to block autophagy activity; but also, the accumulation of p62 in cells treated with BGP. Results showed that choroquine induced autophagosome accumulation (Fig. 3C). p62 was also significantly increased at all incubation times tested, indicating that hyperphosphatemia was reducing the autophagic flux of myoblasts (Fig. 3D). To determine whether ILK was involved in the effect of BGP on mTOR activity and autophagy, we analysed phosphorylation of S6 and p62 accumulation in C_2_C_12_ cells transfected with siILK or with scramble and treated with BGP for 48 h, finding that the increases in pS6 (Fig. 3E) and p62 (Fig. 3F) were prevented when ILK expression was knocked down.

**Figure 5.**
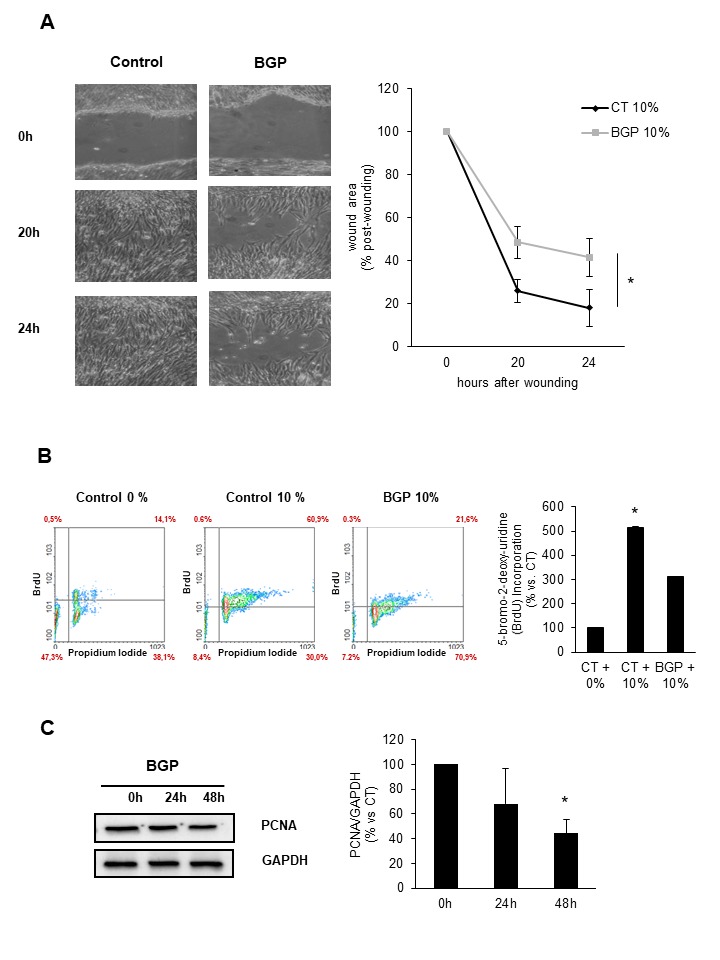
Hyperphosphatemia reduces proliferation of myoblasts **A)** C_2_C_12_ growing in DMEM supplemented with 10% FBS were treated with 10 mM BGP or vehicle for 48 hours. After that, confluent monolayers were scratch wounded. Photographs of wounds were captured at 0, 20 and 24 hours post-wounding to determine the degree of wound closure. A representative experiment is shown. Graphs represent the percentage of control (time 0 h) wound area at different times post-wounding. Results are mean ± SEM from five experiments. * p=0.021 between control and BGP treatment. **B)** C_2_C_12_ were grown in DMEM with (10%) or without (0%) FBS and with 10 mM BGP for 48 hours. BrdU incorporation during the following 24 hours was evaluated by flow cytometry. Bar graph represents the percentage of proliferating cells with respect to the control cells growing in DMEM without FBS. Results are mean ± SEM from six experiments. * p < 0.05 vs control 0%. **C)** C_2_C_12_ cells were grown in DMEM supplemented with 10% FBS and treated with 10 mM BGP for 24 and 48 hours. PCNA expression was evaluated by western blot. A representative blot is shown. Bar graph represents the densitometric analysis of the bands. The results are expressed as a percentage of control and are the mean ± SEM from five different experiments. * p< 0.05 vs control.

**Figure 6.**
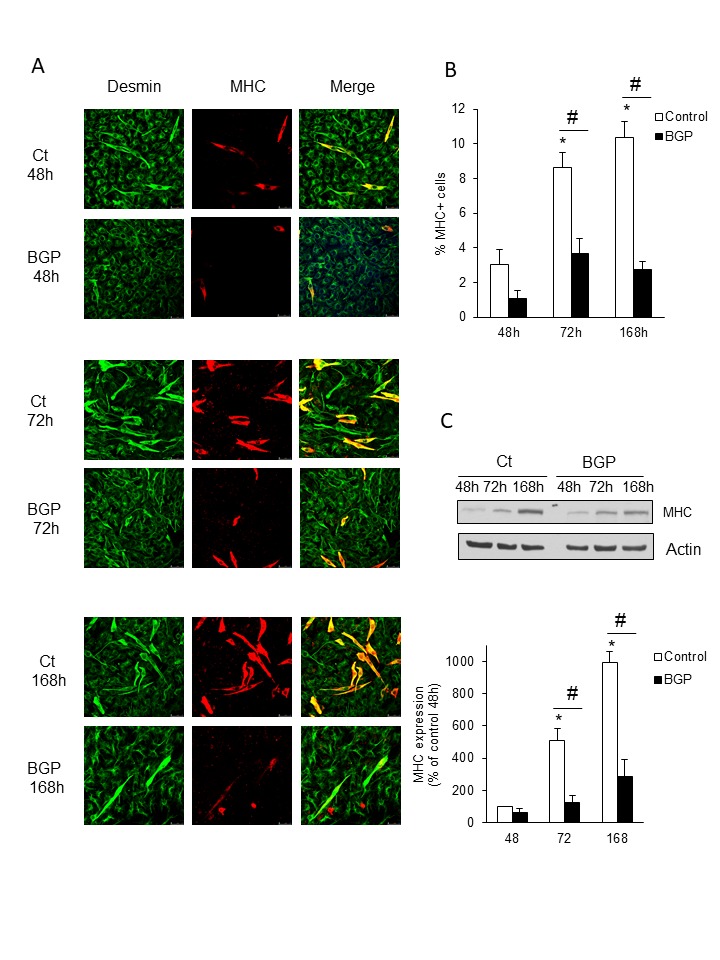
Hyperphosphatemia impairs myogenic differentiation of myoblasts C_2_C_12_ were grown with 2% horse serum for 48, 72 and 168 hours to promote myogenic differentiation. **A)** Myosin heavy chain (MHC, red) and desmin (green) were analysed by immunofluorescence with specific antibodies using a confocal microscopy. **B)** MHC positive cells were counted and represented in a bar graph. Results are mean ± SEM from three experiments. * p< 0.05 vs control 48 h. #p<0.001 vs control at the same time. **C)** MHC expression was evaluated by western blot. A representative blot is shown. Bar graphs represent the densitometric analysis of the bands. Results are mean ± SEM from three experiments. * p< 0.05 vs control 48 h. #p<0.001 vs control at the same time.

### Inhibition of mTOR with rapamycin increases autophagy and protects myoblast from BGP-induced senescence

To analyse whether mTOR activation was determining in BGP-induced senescence, we treated myoblast with 10 mM BGP during 48 h in the presence or the absence of the mTOR inhibitor, rapamycin. As it is shown in Fig. 4A, rapamycin completely abolished mTOR activity assessed by pS6 protein content. In these conditions, also the expected decrease in autophagy promoted by BGP, assessed by the decrease in LC3II/LC3I ratio and the p62 accumulation, was abrogated in the presence of rapamycin (Fig. 4B). In addition, rapamycin prevented the increase in p53 (Fig. 4C) and the increase in SA-β-Gal activity (Fig. 4D) induced by BGP treatment for 48 h, suggesting that senescence induced by BGP depends on mTOR activation and autophagy reduction.

**Figure 7.**
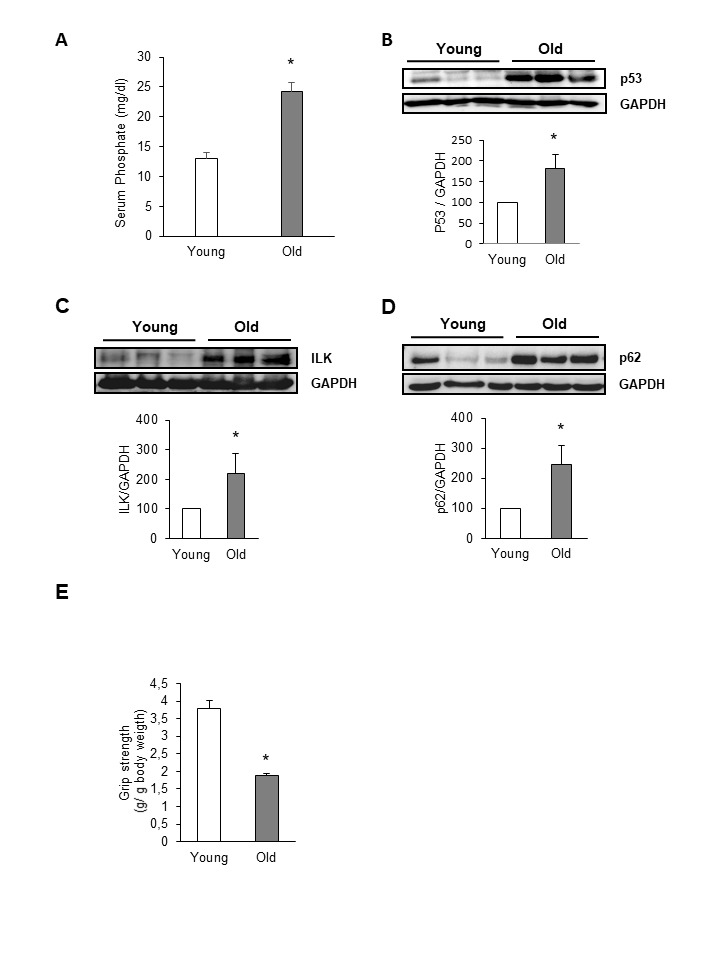
Aged mice present elevated serum phosphate linked to higher expression of ILK and senescence genes and loss of muscle strength Twenty-four-month-old mice (Old) were compared with five-month-old ones (Young). **A)** Serum phosphate concentration was assessed by a colorimetric method. Graph represents the mean ± SEM from values obtained from 10 animals per group. * p< 0.05 vs young. **B-D)** p53 **(B)**, ILK **(C)** and p62 **(D)** expression was evaluated by western blot in protein extracts isolated from vastus from old and young mice. A representative blot is shown in each case. Bar graphs represent the densitometric analysis of the bands. The results are expressed as a percentage of young mice and are the mean ± SEM from ten animals per group. * p< 0.05 vs young. **E)** Forelimb grip strength test was performed in young and old mice. Results are mean ± SEM from ten animals per group. * p< 0.05 vs young.

### Hyperphosphatemia reduces proliferation of cultured myoblasts

We analysed the effect of hyperphosphatemia on the proliferative capacity of myoblast. For this purpose, we performed three different approaches. First, cultured C_2_C_12_ cells were subjected to scratch wound healing assay in the presence or the absence of 10 mM BGP using 10% FBS to stimulate proliferation. We observed a delay in the closure of the wound in cells treated with BGP as compared with cells growing in control conditions (Fig. 5A). Second, we analysed BrdU incorporation into DNA in cultured cells by flow cytometry. We compared cells growing in DMEM without FBS with cells growing in DMEM with 10% FBS in the presence or the absence of 10 mM BGP during 48 h. Results showed that BGP reduced BrdU incorporation significantly compared to cells growing in DMEM with 10% FBS without BGP (Fig. 5B). Finally, we analysed the expression of PCNA (proliferating cell nuclear antigen) in cultured cells growing in DMEM supplemented with 10% FBS and treated with 10 mM BGP at indicated times and we found a progressive decline in PCNA expression in the presence of BGP (Fig. 5C). After that, we concluded that hyperphosphatemia was limiting the proliferative capacity of cultured myoblasts.

### Hyperphosphatemia impairs myogenic differentiation of myoblast

To promote C_2_C_12_ myogenic differentiation cells were growth with 2% horse serum during seven days in the presence or the absence of 10 mM BGP. The expression of MHC was recorded at 48 h, 72 h and 168 h of treatment. As it can see in Fig. 6A and 6B, BGP significantly diminished the number of MHC positive cells indicating a reduction in myotube formation. The expression of MHC was also analysed by western blot (Fig. 6C).

### In vivo evidences for the role of hyperphosphatemia in skeletal muscle senescence

To analyse the in vivo relevance of our findings, aged mice (24-months-old) were tested to determine whether, compared to young mice (5-months-old), phosphate serum was increased coinciding with the presence of senescence markers in skeletal muscle. As it is shown in Figure 7A, phosphate serum concentration was elevated in aged mice compared to young mice. Then, we analysed p53 expression in vastus muscle isolated from old and young mice, finding a significant increase in p53 protein content in the muscle from old mice (Fig. 7B) by comparison with young mice. Besides, ILK expression (Fig. 7C) and p62 accumulation (Fig. 7D) were also increased in vastus muscle from old mice, indicating that the intracellular mechanisms elicited by hyperphosphatemia in myoblasts were also modified in vivo.

**Figure 8.**
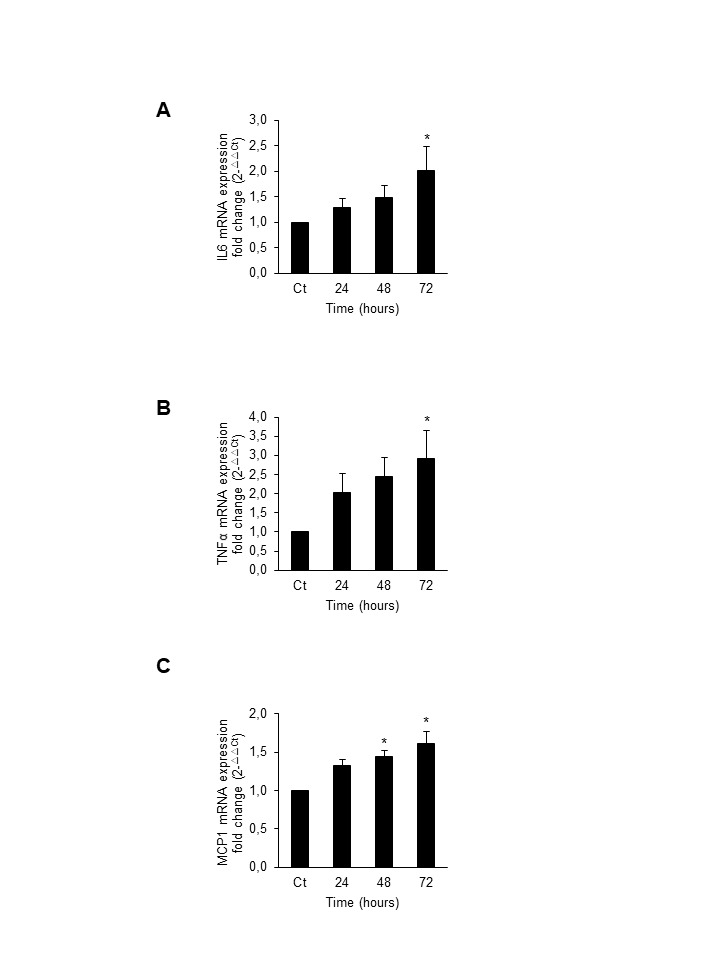
Hyperphosphatemia increases proinflammatory cytokine expression in C_2_C_12_ Cultured C_2_C_12_ were treated with 10 mM BGP for 24, 48 and 72 hours. Total mRNA was isolated and IL6 **(A)**, TNF-α **(B)** and MCP-1 **(C)** mRNA expression was evaluated by RT-qPCR. Results are expressed as fold change respect to control. * p<0.05 vs control

To analyse the impact of hyperphosphatemia and senescence markers in the muscle function, we performed an analysis of the maximal and explosive force production by the forelimb grip strength test in young and old mice. Compared to young mice, the grip strength was significantly lower in all 24-month-old mice (Fig. 7E), whereas no differences were found in body mass and in muscle mass between both groups. Serum phosphate levels were significantly negatively correlated with muscle force (Pearson r=0.52, p=0.0008), suggesting that a cause-effect relationship could be established between hyperphosphatemia and loss of muscle force (Fig. 8).

## DISCUSSION

Sarcopenia is a geriatric syndrome characterized by the loss of muscle function. The factors that initiate and maintain sarcopenic process remain to be elucidated at this moment. In this regard, a role has been described for the increased circulating levels of tumor necrosis factor-alpha (TNF-α), interleukin-6 (IL-6), interleukin-1 (IL-1), and C-reactive protein (CRP) present in the elderly [32] linked with a progressive increase in glucocorticoid and catecholamine levels. Decline in sexual hormones and rise in inflammatory cytokines are also directly associated with muscle mass loss and muscle strength reduction in the elderly [33]. We propose here hyperphosphatemia as one of the systemic factors involved in the genesis of sarcopenia.

Sarcopenia is accompanied by a significant decline in satellite cell function and cell number, which compromises the regenerative capacity of aged muscle [5]. It has been proposed that satellite cells from old mice exposed to a young environment can be rejuvenated [34], indicating that there are systemic factors inducing the dysfunction found in satellite cells with advanced age. Our results demonstrate that hyperphosphatemia induces cellular senescence in cultured myoblast and also reduces proliferative capacity and the myogenic differentiation which are probably mechanisms involved in the loss of muscular mass and strength.

The increase in serum phosphate levels has been related to aging and to the increase in morbi-mortality in chronic kidney disease patients [35]. Muscle wasting is present in both, aging and chronic kidney disease patients [36]. Serum phosphate levels inversely correlate with the lifespan of diverse species [37] and hyperphosphatemia has been linked with premature aging phenotype in Klotho deficient mice and in FGF23 KO mice [19, 20]. In vitro studies have demonstrated that the increase in extracellular phosphate concentration modifies the behaviour of cultured cells by inducing osteogenic and osteoclastic differentiation or cellular senescence in smooth muscle cells [21, 17]. It is the first time that the potential role of hyperphosphatemia in modifying myoblast cell cycle has been shown. To perform the experiments, we used murine C_2_C_12_, the most commonly used myogenic cell line. Due to the difficulty to culture and maintain primary satellite cells, C_2_C_12_ cells have been extensively used since their cell signalling responses remain sufficiently similar to those of primary embryonic and adult myoblasts [38]. On the other hand, C_2_C_12_ cells have been widely used in cellular senescence related studies [39]. C_2_C_12_ cells were exposed to high extracellular phosphate concentration by adding the phosphate donor BGP, as previously described [17, 40]. To assess whether phosphate entered the myoblast to perform its effect, we used a specific inhibitor of the Na-Pi cotransporter termed PFA [41]. We found that cells did not undergo to senescence when the phosphate intake was blocked.

Cellular senescence was assessed by the increased expression of several cell cycle regulators related to senescence: acetylated p53, p53 and p21, and also by the increase in the SA-β-Gal activity, without finding changes in p16 expression. Cellular senescence promoted without p16 increment has been previously described [16]. An increased accumulation of p21 and p53 proteins has been previously described in satellite cells from old animals compared to young animals, contributing to a lower proliferation rate of these cells [42]. Cellular senescence has been identified as a mechanism involved in the genesis of the myopathy associated with muscular dystrophy mouse model [43] and related to premature muscle wasting in young mice deficient in the protein Bmi1 [44] or mutant mice carrying BubR1 hypomorphic alleles [45].

Exploring the intracellular mechanisms involved in phosphate-induced senescence of myoblasts, we found that ILK plays a critical role. ILK has a broadly expression in muscle cells. In cardiomyocytes, ILK modulates contraction [46] and, in skeletal muscle cells, it is involved in muscle hypertrophy [47], protects fibres from stress-induced damage [48] and resembles muscular dystrophy when deleted [49]. However, our results demonstrate that overexpression of ILK induced by hyperphosphatemia leads to cellular senescence. ILK has been previously related to aging [27-28] and we report here that ILK plays an essential role in senescence induced by extracellular high phosphate concentration in cultured myoblasts. ILK expression and activity, assessed by GSK3β phosphorylation [50], increased after phosphate addition, and the down regulation of ILK gene by small interfering RNA protected cells from phosphate-induced senescence.

ILK is connected with numerous intracellular signalling pathways, such as the mammalian target of rapamycin (mTOR), a serine-threonine kinase, which plays an essential role in autoimmune disorders, obesity and aging [51]. We demonstrate that hyperphosphatemia induces a persistent mTOR activation, measured as the phosphorylation of ribosomal S6 protein, the substrate of p70 S6 Kinase [52], indicative of increased activation of mTOR complex1 (mTORC1), in an ILK dependent way. Persistent activation of mTOR is related to cancer, diminished cardiac performance and obesity associated metabolic diseases and has been associated with a significant reduction in autophagy [53]. Autophagy is an evolutionary conserved catabolic process, by which cytoplasmic proteins and organelles are engulfed in autophagosomes and degraded after fusion with lysosomes, preventing waste accumulation [54]. Hyperphosphatemia induces the accumulation of p62 protein and reduces the ratio LC3II/LC3I, both indicative that autophagic flux is reduced [55]. The changes in p62 and in the LC3II/LC3I ratio were inhibited with rapamycin, indicating that the reduction in autophagy was dependent on the activation of mTOR by hyperphosphatemia. Reduced autophagy induced cellular senescence in primary fibroblasts [56] and in quiescent satellite cells [57]. We demonstrate that the hyperphosphatemia is not able to induce senescence in the presence of rapamycin, indicating that the activation of mTOR and the reduction in autophagy are essential in the effect of high phosphate on senescence.

As a consequence of cellular senescence induced by hyperphosphatemia, cultured myoblasts lose their proliferative capacity and this phenomenon has been related to the development of sarcopenia. In this regard, it has been described that myostatin and activin, which are linked to sarcopenia, are also inhibitors of the myoblasts proliferation [58].

To reinforce the relevance of the effect of hyperphosphatemia in loss of skeletal muscle, we searched for evidences of senescence markers in the vastus of old mice and found a higher expression of p53 in muscle from old mice together with higher ILK and p62 expression, resembling the results obtained in cultured myoblast exposed to high extracellular phosphate concentration. Old mice showed a higher concentration of phosphate in serum compared to young mice. Although, a direct link between hyperphosphatemia and senescence sings in muscular tissue could not be stablished with present results, interestingly, we found that those mice with higher phosphate levels have less muscle strength in their forelimbs, measured by the grip strength test, a widely used method to assess skeletal muscle function in rodents. Old mice showed a reduction in muscle strength of about 40% compared to young mice, indicating sings of sarcopenia. In conclusion, we propose that hyperphosphatemia associated with aging could be an important inductor of sarcopenia. Our results show that high extracellular phosphate concentration induces overexpression of ILK that provokes mTOR activation and autophagy reduction, promoting cellular senescence of cultured myoblasts and reducing their proliferative capacity. This could be a mechanism involved in the development of sarcopenia associated with aging since we found loss of muscle strength in old mice in parallel with higher serum phosphate levels and expression of senescence markers, such as ILK, p53 and p62, resembling the mechanism observed in the i*n vitro* studies. Further experiments will be necessary to demonstrate a direct cause-effect relationship between hyperphosphatemia and sarcopenia.
